# Amorphous Carbon Coatings for Total Knee Replacements—Part II: Tribological Behavior

**DOI:** 10.3390/polym13111880

**Published:** 2021-06-05

**Authors:** Benedict Rothammer, Max Marian, Kevin Neusser, Marcel Bartz, Thomas Böhm, Sebastian Krauß, Stefan Schroeder, Maximilian Uhler, Simon Thiele, Benoit Merle, Jan Philippe Kretzer, Sandro Wartzack

**Affiliations:** 1 Engineering Design, Friedrich-Alexander-University Erlangen-Nuremberg (FAU), Martensstr. 9, 91058 Erlangen, Germany; kevin.k.neusser@fau.de (K.N.); bartz@mfk.fau.de (M.B.); wartzack@mfk.fau.de (S.W.); 2 Forschungszentrum Jülich GmbH, Helmholtz-Institute Erlangen-Nürnberg for Renewable Energy, Cauerstr. 1, 91058 Erlangen, Germany; t.boehm@fz-juelich.de (T.B.); si.thiele@fz-juelich.de (S.T.); 3 Department of Materials Science & Engineering, Interdisciplinary Center for Nanostructured Films (IZNF) Institute I, Friedrich-Alexander-University Erlangen-Nuremberg (FAU), Cauerstr. 3, 91058 Erlangen, Germany; sebastian.s.krauss@fau.de (S.K.); benoit.merle@fau.de (B.M.); 4 Laboratory of Biomechanics and Implant Research, Clinic for Orthopedics and Trauma Surgery, Heidelberg University Hospital, Schlierbacher Landstr. 200a, 69118 Heidelberg, Germany; stefan.schroeder@med.uni-heidelberg.de (S.S.); maximilian.uhler@med.uni-heidelberg.de (M.U.); philippe.kretzer@med.uni-heidelberg.de (J.P.K.); 5 Department of Chemical and Biological Engineering, Friedrich-Alexander-University Erlangen-Nuremberg (FAU), Egerlandstr. 3, 91058 Erlangen, Germany

**Keywords:** DLC coating, biomedical applications, biotribology, UHMWPE, CoCr, Ti64, total knee arthroplasty, pin-on-disk, friction, wear

## Abstract

Diamond-like carbon coatings may decrease implant wear, therefore, they are helping to reduce aseptic loosening and increase service life of total knee arthroplasties (TKAs). This two-part study addresses the development of such coatings for ultrahigh molecular weight polyethylene (UHMWPE) tibial inlays as well as cobalt-chromium-molybdenum (CoCr) and titanium (Ti64) alloy femoral components. While the deposition of a pure (a-C:H) and tungsten-doped hydrogen-containing amorphous carbon coating (a-C:H:W) as well as the detailed characterization of mechanical and adhesion properties were the subject of Part I, the tribological behavior is studied in Part II. Pin-on-disk tests are performed under artificial synovial fluid lubrication. Numerical elastohydrodynamic lubrication modeling is used to show the representability of contact conditions for TKAs and to assess the influence of coatings on lubrication conditions. The wear behavior is characterized by means of light and laser scanning microscopy, Raman spectroscopy, scanning electron microscopy and particle analyses. Although the coating leads to an increase in friction due to the considerably higher roughness, especially the UHMWPE wear is significantly reduced up to a factor of 49% (CoCr) and 77% (Ti64). Thereby, the coating shows continuous wear and no sudden failure or spallation of larger wear particles. This demonstrated the great potential of amorphous carbon coatings for knee replacements.

## 1. Introduction

The endoprosthetic replacement of the knee joint aims at restoring its functionality, which has been reduced due to gonarthrosis and rheumatoid arthritis, enabling patients to enjoy a mobile and pain-free life [1]. The numbers of this surgical treatment continue to grow, and the implantation of total knee arthroplasties (TKAs) is becoming relevant for increasingly younger patients [2,3]. Thereby, several implant types/designs are available and chosen depending on severity or spread of the osteoarthritis, and ligament stability [4,5]. Essentially, TKAs incorporate a hard-on-soft pairing consisting of a femoral component rubbing against the medial and lateral bearing surfaces of a tibial plateau. For the femoral component, oxide ceramics or cobalt-chromium-molybdenum (CoCr) alloys are usually used. With regard to the high hardness and risk of sudden fracture-like failure of ceramic knee prosthesis with subsequent joint immobility, the use of more damage-tolerant, metallic femoral components is predominant [6,7,8]. Despite tending to exhibit inferior tribological behavior, titanium (Ti64) alloys are gaining increasing attention due to enhanced biocompatibility [5]. In addition, mostly ultrahigh molecular weight polyethylene (UHMWPE) or to a growing extent highly cross-linked polyethylene (HXLPE) are employed for the tibial inlay [5,9]. Moreover, the contacts between the femoral and tibial components are lubricated by synovial fluid (SF), leading to a full or partial separation of the contacting surfaces [10,11]. Although the joint experiences dynamic and complex motions and stresses, the service life of TKAs can be up to 25 years for a majority of the patients [12,13]. However, aseptic loosening and periprosthetic osteolysis are still a major cause for premature implant failure [14,15]. This has a multifactorial etiology [16], with wear particles from the implant materials being largely implicated [17,18,19]. Accordingly, the tribological behavior of TKAs has a substantial influence on the service life in the human body [20]. Thereby, novel implant designs [4], material combinations [5] or surface technologies, such as texturing [21] or coating, in order to reduce wear, are the subject of current research efforts. In particular, the application of thin coatings can extend the service life of implants and prevent patients from having to undergo early revision surgery. Specifically, titanium-nitride- [22], titanium-carbide- [23], zirconium- [24], tantalum- and amorphous carbon-based [25] coatings have already been investigated in the context of large joint implants [26]. Diamond-like carbon (DLC) coatings can be considered particularly attractive due to their advantageous ratio of hardness-to-elasticity and the ability to form wear-protecting transfer layers [27,28]. These excellent and tunable mechanical properties [29] can be complemented by medically relevant properties [30], such as chemical resistance, antibacterial behavior and good biocompatibility [31,32].

There are several studies on DLC-coated total hip arthroplasties (THAs). Using a hip simulator as well as pin-on-disk model tests under lubrication with a 1 wt.% NaCl water solution, Tiainen [33] reported a 10^5^–10^6^-fold wear reduction of the femoral head through a hydrogen-free, tetrahedral amorphous carbon coating (ta-C) compared to an uncoated hard-on-soft or hard-on-hard contact. In addition, a 10-times reduction of the wear rate of the plastic acetabular cup was achieved. In contrast, Saikko et al. [34] found no significant differences in the wear behavior of alumina, CoCr and DLC-coated CoCr femoral heads using a biaxial hip simulator and diluted calf serum (CS) as a lubricant. Similarly, Scholes et al. [35] reported higher friction from a DLC coating of stainless steel femoral heads in contact against a UHMWPE cup in a hip function simulator under lubrication with aqueous carboxy methyl cellulose solution (CMC), which was mainly attributed to a higher surface roughness. Regarding wear, there were no clear results and strong variations between the coated specimens. However, Ortega-Saenz et al. [36] in turn demonstrated significantly improved wear behavior for DLC coatings compared to uncoated references and a CrN coating. Their performance was comparable to a TiN/CrN multilayer coating. This was the case for the coated CoCr femoral head as well as the acetabular cup. Thereby, the hip simulator was lubricated with an artificial SF based upon diluted bovine serum (BS). Regarding TKAs, fewer studies on the effects of DLC-coatings on the tribological behavior can be found. This is probably due to the more complex geometries. Merely, Oñate et al. [37] used a knee wear simulator under distilled water lubrication and obtained a five-fold decrease of the UHMWPE wear by a DLC-coating on the CoCr femoral component compared to uncoated references.

In addition to these application-oriented tests, a number of investigations were carried out with simplified disk-on-disk, cylinder-on-disk or pin-on-disk model setups. As such, Lappalainen et al. [38] reported improved corrosion resistance and a 30–600-fold reduction of the wear rate when depositing ta-C coatings on CoCr or Ti64 alloy pins when sliding against UHMWPE disks under lubrication with a 1 wt.% NaCl water solution. Conversely, Sheeja et al. [39,40] found no significant improvement regarding the wear behavior of UHMWPE sliding against multilayer ta-C coated CoCr under water or simulated body fluid lubrication. Loir et al. [41] performed pin-on-disk tests under dry conditions and very high pressures compared to actual hip implants. Thereby, excellently adhering ta-C coatings on the 316L stainless steel fabricated by femtosecond pulsed laser ablation were able to significantly reduce the wear rates. Reciprocal ball-on-disk tests with SiC balls under dry conditions by Xie et al. [42] also demonstrated good wear resistance of hydrogenated amorphous carbon films deposited on UHMWPE by plasma-enhanced chemical vapor deposition (PECVD). Choudhury et al. [43] encountered adverse frictional and wear behavior of DLC-coated Al_2_O_3_ ceramic disks rubbing against an UHMWPE cylinder in reciprocating sliding lubricated by diluted BS. Guo et al. [44] investigated the influence of load and lubricant on DLC coatings in a CoCr pin-on-CoCr disk setup under reciprocating motion and lubrication with a bovine serum albumin solution (BSA) as well as physiological saline (PS). For the latter, it was reported that a DLC transfer layer was formed, leading to low friction and good wear protection. This was not the case for BSA lubrication, where the boundary absorption layer of proteins could be considered as a main influencing factor. Accordingly, experiments from Corona-Gomez et al. [45] with chemical vapor deposited (CVD) DLC coatings on CoCr disks rubbing against UHMWPE balls under lubrication with distilled water revealed friction and wear reduction. Lately, Rothammer et al. [46] used a metal ring-on-UHMWPE disk tribometer in rotational sliding mode and lubrication by bovine calf serum (BCS) to screen various types of DLC coatings, namely pure, silicon-oxide- and titanium-doped hydrogenated amorphous carbon coatings deposited on CoCr as well as Ti64. Thereby, especially the pure and silicon-oxide-doped DLC coatings represented promising systems for increasing the wear resistance of implants compared to the uncoated references. However, significant UHMWPE wear was still observed, partly due to the high hardness and roughness of the coated counter-bodies.

Apparently, in vitro tribological tests to evaluate the wear behavior and service life of TKAs differ in terms of complexity and transferability to in vivo conditions. In this context, the contact conditions play a decisive role and are determined by the lubrication as well as the test setup. Thereby, knee wear simulators according to ISO 14243 [47,48,49] represent key setups with real components. Testing is mostly performed for typical load cases and in a closed chamber with a substitute SF tempered to 37 ± 1 °C. Usually, 5 × 10^6^ cycles are carried out and the wear behavior as well as particle number, size, morphology and their cytological effect are investigated [50,51], showing satisfactory agreement with phenomena observed in vivo. However, these approaches also involve extensive experimental efforts, especially for the screening and analysis of novel material combinations and coatings. This can be reduced to some extent, for example, by a slight simplification to rolling-sliding test-rigs for knee components [52]. Furthermore, pin-on-disk configurations are prominent model tests. With simplified geometries and pure sliding conditions only, these are more remote from the actual conditions but ideal for investigating isolated wear mechanisms and for comparing wear rates [51]. Thus, tribometer tests are particularly useful for evaluating the tribological effectiveness of coating systems and to qualitatively compare the findings to existing literature and experiments closer to the actual application. However, there are no established or specified standards with respect to testing in the context of TKAs. Hence, it has to be ensured that the experimental conditions of the model pairing still guarantee sufficient relevance and transferability.

Numerical modeling can be utilized for the analysis of the test conditions and the influence of the coatings on relevant properties as well as for a comparison of a model pin-on-disk contact with contact situations under more realistic settings [53]. Contact simulations based upon the finite element method (FEM) have been widely applied to study the stresses and predict the wear evolution in TKAs [54,55,56,57]. Kang et al. [58] even numerically analyzed the influence of different surface properties and coatings on the calculated weight loss and wear depth. Thereby, a DLC-coated femoral component was reported to feature inferior behavior compared to uncoated CoCr or other types of coatings. However, this was subject to a number of assumptions regarding wear coefficients and surface roughness. Furthermore, despite its decisive role, lubrication was neglected or strongly simplified in most theoretical studies on TKAs. Early approaches to tackle this issue neglected the local elastic deformation of the contacting bodies or assumed strongly simplified kinematics and geometries [59,60,61,62,63]. However, with advances in the simulation of the elastohydrodynamic lubrication (EHL) in THAs, some more sophisticated numerical models have also been developed for TKAs. The studies from Su et al. [64,65] based upon a coupled solution of the hydrodynamics and the elastic deformation by a multigrid finite difference and constraint column model or Gao et al. [66] using a multigrid and spherical fast Fourier transformation-based approach may be mentioned as examples. Lately, Marian et al. [67] introduced an approach based upon full-system FEM combined with comparisons with experimental measurements from a knee simulator using optical fluorescence microscopy [68]. Thus, in principle, some numerical approaches are available. However, the impact of coatings on the lubrication conditions has not been evaluated yet in the aforementioned works.

To summarize, amorphous carbon coatings offer the potential to reduce implant wear and thus to increase the service life of TKAs. However, contradictory results have been reported in literature, with DLC coatings either leading to an improvement or a deterioration in friction and/or wear behavior. Here, the coating composition and deposition process, the adhesion to the substrate and the surface topography [69] as well as the testing conditions and the lubricant [70,71] can be considered to be most relevant for the tribological performance [72]. Due to occasionally insufficient description and differences in the mentioned aspects between the various studies as well as to the real knee implant, the suitability of DLC coatings cannot be evaluated conclusively yet. Therefore, this contribution aims at reproducible and, most importantly, transferable findings on wear-reducing DLC coating systems specifically for CoCr and Ti64 femoral as well as UHMWPE tibial components of knee replacements. This is accomplished by a thorough description of all applied methods and a unique combination of numerical simulation and experimental testing. The deposition and detailed characterization in terms of biochemical, mechanical and adhesion properties were the subject of Part I [73]. Thereby, tungsten-doped hydrogen-containing amorphous carbon coatings (a-C:H:W) were deposited on CoCr and Ti64, while a pure hydrogenated amorphous carbon coating (a-C:H) was applied to UHMWPE. The coatings exhibited a significant increase in indentation hardness compared to the substrates and featured a sufficiently high coating adhesion for metal specimens and for UHMWPE, respectively. Building on this, the tribological behavior is examined in this contribution (Part II). To analyze the fundamental wear behavior and mechanisms under consistent conditions and with a minimum of distorting factors, model tests with CoCr and Ti64 pins on UHMWPE disks are performed using an environmentally-controlled tribometer. Numerical EHL modeling is utilized to assess if the experimental conditions are representative for TKAs and to evaluate the influence of the coatings on the contact and lubrication conditions. In the long-term tribometer tests, the frictional behaviors are analyzed in-situ while the wear performance is characterized ex-situ using light microscopy (LM) and laser scanning microscopy (LSM). Scanning electron microscopy (SEM), Raman spectroscopy and particle analyses are also employed to investigate the wear behavior.

## 2. Materials and Methods

First, the studied materials and coatings will be described briefly. This is deliberately kept short as they have already been introduced and analyzed in detail in Part I of this study [73]. Secondly, the setup and procedure of the experimental tests on the pin-on-disk tribometer will be explained. Thirdly, the methods for the ex-situ characterization of the wear behavior are explained. Finally, the accompanying numerical models of the initial lubricating conditions are described.

### 2.1. Materials

Disks made from UHMWPE [74] (Chirulen^®^ GUR 1020, Vreden, Quadrant EPP, Germany) with a diameter of 45 mm, a height of 8 mm and a central beveled pass-through hole with a diameter of 6.6 mm for fixation were the main bodies of this investigation. Co28Cr6Mo (CoCr [75], Peter Brehm, Weisendorf, Germany) and Ti6Al4V ELI wrought alloy (Ti64 [76], Jäckel + Co. Edelstahl Metalltechnik, Schöneck, Germany) pins with a diameter of 10 mm and a head radius *R* of 100 mm (spherical ending) were used as counter-bodies. An a-C:H:W coating was applied to the CoCr (CoCr:W) and Ti64 (Ti64:W) pins and an a-C:H coating to the UHMWPE (UHMWPE:H) disks by physical vapor deposition (PVD). A thin adhesive layer of chromium (Cr) and an intermediate layer of tungsten carbide (WC) were used to achieve a high coating-substrate adhesion. In contrast, the a-C:H coating was applied directly to the polymeric substrate by reactive PVD without intermediate layers. The characterization of the mechanical and adhesion properties was reported in detail in Part I of this study [73]. For the convenience of the reader, only the mechanical key parameters (averaged values) that are also relevant for the numerical modeling and the discussion in Part II are summarized in Table 1.

### 2.2. Tribological Testing

**Figure 1:**
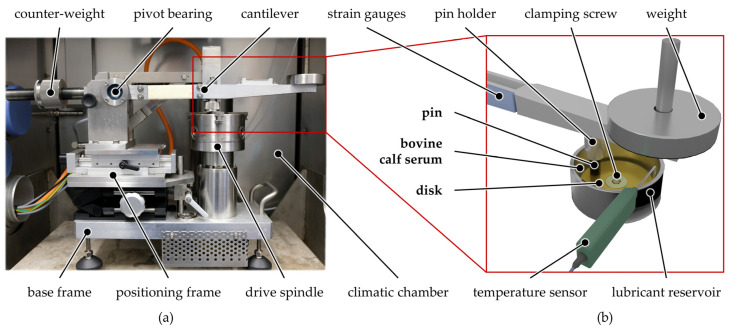
Photograph of the pin-on-disk tribometer inside the climatic chamber (**a**) and schematic representation of the experimental setup (**b**).

Tribological testing was carried out using a pin-on-disk tribometer (K-SST, KTmfk, Erlangen, Germany) as illustrated in Figure 1 in rotational sliding mode. Thereby, the disk was clamped in the stainless-steel lubricant reservoir with a screw and was driven by a three-jaw chuck.

To lubricate the contact, the reservoir was filled with 25 mL of a substitute SF (Laboratory for Biomechanics and Implant Research, Clinic for Orthopedics and Trauma Surgery, Heidelberg University Hospital, Heidelberg, Germany). Therefore, BCS (Biochrom, Berlin, Germany) with a composition according to Table 2 was used and diluted to a protein content of 20 ± 1 g/L according to ISO 14234-1 [47] by adding deionized water. For an anti-microbiological function and to reduce calcium phosphate layers on the material surfaces, 1.85 g/L sodium azide (NaN_3_) as well as 5.85 g/L ethylenediaminetetraacetate (EDTA) were added. Since the fluid substantially influences the tribological performance, a detailed characterization of the respective rheological properties and the suitability for mimicking human SF was already shown and discussed in [77]. The fluid was pre-cooled to 4 °C and subsequently deep-frozen at −20 °C. Prior to testing, the BS was naturally thawed to 8 °C.

The pin was fixed to a cantilever, and a normal force *F* of 10 N was applied by means of a weight. The friction force was determined with strain gauges based upon the deflection of the cantilever with a scanning rate of 3 Hz. Thus, the coefficient of friction (COF) could also be determined from the quotient of the frictional force and the normal load. With a disk rotational speed of 1 s^−1^ and a wear track radius of 15.92 mm, the sliding velocity *u* was set to 0.1 m/s. The tribometer was placed on a vibration damping frame and in an environmental chamber (3436/15, Feutron, Langenwetzendorf, Germany). A constant temperature of 37 ± 0.2 °C could thus be ensured in the lubricant bath and an ambient relative humidity of 50% in the surrounding environmental chamber. Uncoated references as well as coated pairings were tested, more specifically the following four disk|pin combinations:

- UHMWPE|CoCr
- UHMWPE|Ti64
- UHMWPE:H|CoCr:W
- UHMWPE:H|Ti64:W

For each pairing, three repetitions with new specimens were performed for statistical purposes. The experimental duration covered an overall sliding distance *s* of 2 × 10^4^ m, corresponding to 2 × 10^5^ cycles. To examine the temporal wear evolution as well, the tests were divided into 10 intervals of 2 × 10^3^ m or 2 × 10^4^ cycles, respectively. After each interval, the specimens were cleaned non-tactilely with distilled water and characterized regarding wear-related changes as described in Section 2.3. Subsequently, the evaporated or absorbed substitute SF was refilled to a level of 25 mL and the next interval was pursued.

### 2.3. Wear Characterization

To quantitatively evaluate the wear behavior of the pins and disks, the specific circumstances of the setup, the materials as well as the wear phenomena of the specimens had to be considered. Gravimetric or volumetric measurements were not suitable because of soaking effects due to the fully flooded conditions, especially for the UHMWPE disks [72]. Therefore, the wear characterization was mainly done by digital light microscopy (LM; DM4000, Leica Microsystems, Wetzlar, Germany). After each test interval, the surfaces of the pins as well as the wear track on the disks were imaged at four 90° shifted positions. In addition, 3D measurements of height were carried out at the same positions using laser scanning microscopy (LSM; VK-X200, Keyence, Osaka, Japan) at the end of the test series and for one pairing per type after each interval. Subsequently, quantitative wear rates were calculated as:

(1) $$k=\;\frac{W_{V}}{F\;\cdot \;s}$$

where volumetric wear *W*_v_ was determined from the planimetric wear *W*_p_ in cross-section multiplied by the circumference of the wear track *c*. For the uncoated reference disks, this was done by means of the summation of adhesive wear *W*_p,adh_ above and abrasive wear *W*_p,abr_ below the centerline of the surrounding specimen surfaces:

(2) $$W_{V,\;\mathrm{ref}.\;\mathrm{disk}}{=W}_{p}\;\cdot \;c=\left(W_{p,\mathrm{adh}}{+W}_{p,\mathrm{abr}}\right)\;\cdot \;c.$$

The coated disks showed less pronounced abrasion and adhesion but distinct plastic deformation at the edges of the micro-ploughed wear tracks *W*_p,AB_. Therefore, the *f*_AB_ parameter from Czichos [78] was employed and the material accumulations *W*_acc_ were taken into account in the calculation of the volumetric wear:

(3) $$W_{V,\;\mathrm{coated}\;\mathrm{disk}}{=f}_{\mathrm{AB}}\;\cdot {\;W}_{p,\mathrm{AB}}\;\cdot \;c=\left(W_{p,\mathrm{AB}}{\;-\;W}_{\mathrm{acc}}\right)\;\cdot \;c.$$

The wear analysis of the pins was performed by evaluating the resulting wear calottes and scores. Thereby, the volumetric wear of the coated pins was calculated as the difference of the pin curvature and the worn off coating thickness:

(4) $$\begin{array}{cc} W_{V,\;\mathrm{coated}\;\mathrm{pin}\;}= & \sum_{i=1}^{n}\left[\frac{\pi }{3}\cdot \left(R_{i}\pm \sqrt{R_{i}^{2}-r_{c,i}^{2}}\right)\cdot \left(r_{c,i}^{2}+R_{i}\cdot \left(R_{i}\pm \sqrt{R_{i}^{2}-r_{c,i}^{2}}\right)\right)\right]\\ \; & -\left[\frac{\pi }{3}\cdot \left(R_{i}\pm \sqrt{R_{i}^{2}-r_{c,i}^{2}}-t_{c,i}\right)\cdot \left(r_{c,i}^{2}+R_{i}\cdot \left(R_{i}\pm \sqrt{R_{i}^{2}-r_{c,i}^{2}}-t_{c,i}\right)\right)\right]. \end{array}$$

where corresponding radii is *R*_i_, the curved calotte radii *r*_c,i_ and the worn off coating thickness *t*_c,i_. The analysis of the reference pins was constrained by the fact that no clear and uniform calotte was worn off the head radius. Instead, the large deformation of the UHMWPE mating body was found to extend the wear over a wider area, including a slight change in the original curvature of the pin surface. Therefore, the wear was approximated by means of summing up the volumes of the developed grooves, scores and shape deviations:

(5) $$W_{V,\;\mathrm{unccoated}\;\mathrm{pin}}=\overset{n}{\underset{i=1}{\sum }}\;\frac{A_{c,i}}{h_{s,i}}\;\cdot {\;R}^{2}\;\cdot \;\arcsin\left(\frac{l_{s,i}}{2\;R}\right)\;-\;\frac{l_{s,i}\;\cdot \;\left(R-h_{s,i}\right)}{2},$$

where cross-sectional areas are *A*_c,i_, the corresponding lengths *l*_s,i_ and heights *h*_s,i_ of the grooves, scores or shape deviations. Furthermore, SEM images and Raman spectroscopy were done to further elaborate the wear phenomena and mechanisms. Raman spectra were recorded by a confocal Raman microscope (WITec alpha300, WITec, Ulm, Germany) with excitation at 457 nm and a laser power of 0.15 mW using a ×100/0.9 NA objective. The spectra were integrated for 2 s with 5 accumulations and background-corrected using the shape-based algorithm in WITec Project FIVE+. After normalization to the maximum signal intensity, mean spectra from up to three different spots at pristine, partially degraded and degraded positions of the specimens outside, at the edge and inside the wear track were determined. In addition, SEM micrographs were taken of the worn surfaces as well as in cross-sections using focused ion beam (FIB) milling (Helios NanoLab 600i, FEI Thermo Fisher, Hillsboro, OR, USA). To this purpose, an acceleration voltage of 5 kV, an electron current of 0.69 nA and working distances ranging from of 4.0 to 5.7 mm were used. Imaging of the coatings’ cross-sections was done at a tilting angle of 40°.

### 2.4. Particle Analysis

In order to characterize the wear particles in the test fluid with respect to their size and morphology, particle analysis according to ASTM F1877-16 [79] was performed. Two intervals of the tribometer tests (Numbers 3 and 7) of each pairing were used for analysis. The test specimens were prepared by acid digestion according to [50,80], filtered through aluminum-oxide filters with a pore-size of 0.1 µm (AnodiscTM 13, WhatmanTM, GE Healthcare Life Sciences, Amersham, UK) and analyzed using high resolution field emission gun scanning electron microscopy (FEG-SEM, LEO 1530, Leo, Oberkochen, Germany) at a magnification of ×10,000. For particle analysis, digital image software (Particleanalyzed_HD, LBI, Heidelberg, Germany) was used [81]. Two filtrations were carried out per interval of each group and three images were made per filter. The analyzed parameters were equivalent circle diameter (ECD), aspect ratio (AR) and roundness (R). In order to detect any changes in the particle characteristics over time, the results of Interval 3 were compared to the results of Interval 7 using a *t*-test for dependent groups. Interval 7 represented the phase after the run-in and was used for group comparison. Therefore, an ANOVA for independent testing and a Bonferroni post-hoc analysis was performed.

### 2.5. Numerical Modeling

Since the lubrication conditions have a direct influence on the friction and wear behavior, two objectives are pursued by numerical modeling. First, to ensure that the experimental conditions in the model test on the tribometer are relevant and representative for TKAs. Secondly, to investigate how the coatings on the pin and the disk affect the pressure and lubricating film formation. Here, a numerical 3D EHL model based upon the full-system FEM approach [82,83] was employed and adapted to fit for the initial conditions of the present experimental configuration. Numerical modeling was already presented in great detail and validated for TKAs in [67,68]. Therefore, only the most important features and differences to the previously published work are described in the following.

The SF’s hydrodynamics was described by a quasi-stationary Reynolds equation:

(6) $$\frac{\partial }{\partial x}\left(\frac{\rho \cdot h^{3}}{12\cdot \eta }\frac{\partial p}{\partial x}\right)+\frac{\partial }{\partial y}\left(\frac{\rho \cdot h^{3}}{12\cdot \eta }\frac{\partial p}{\partial y}\right)-\frac{\partial }{\partial x}\left(\theta \cdot \rho \cdot h\frac{u}{2}\right)=0$$

in slightly modified notation, with the hydrodynamic pressure *p*, the lubricant gap *h*, the density *ρ*, the viscosity *η* as well as the cartesian coordinates *x* and *y* [84]. Cavitation effects were considered by a mass-conserving penalty formulation of the fractional film content *θ* [85]. Shear thinning behavior was accounted for by means of a Cross model [86]:

(7) $$\eta =\eta_{\infty }+\frac{\eta_{0}-\eta_{\infty }}{1+{\left(\alpha \cdot \dot{\gamma }\right)}^{m}}$$

where we assume constant density, viscosity and shear rate $\dot{\gamma }$ in the film height (z-) direction [87]. The corresponding properties of the artificial SF at 37 °C were determined in [77] and are summarized in Table 3.

The fluid film height equation:

(8) $$\;h\left(x,\;y\right){=h}_{0}+\frac{x^{2}}{2\cdot R}+\frac{x^{2}}{2\cdot R}{+\delta }_{1}\left(x,\;y\right){+\delta }_{2}\left(x,\;y\right)$$

was comprised by the distance of the undeformed bodies in the contact center *h*_0_ as well as their geometrical approximation with the pin head radius *R* and the elastic deformations of the disk *δ*_1_ and the pin *δ*_2_. The latter were calculated by applying the linear elasticity equation:

(9) ∇σ=0

with the stress tensor:

(10)  σ=C · ε(U)

following Hooke’s law, where *C* is the compliance matrix, *ε* the strain tensor and *U* the displacement vector. Thus, the deformation results from the local displacement in z-direction:

(11) $$\;\delta \left(x,\;y\right)=\;\left|U_{z}\left(x,\;y\right)\right|.$$

**Figure 2:**
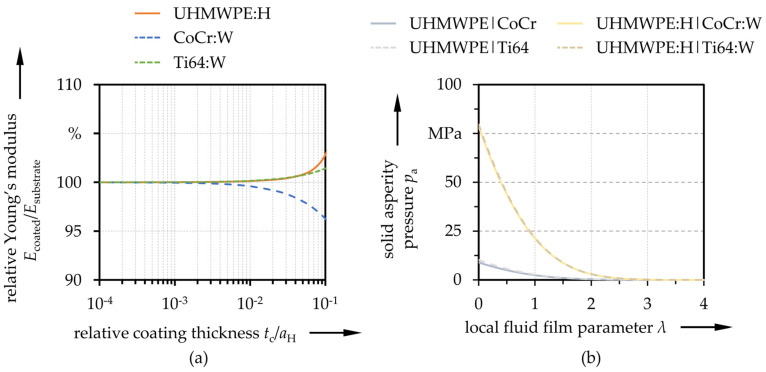
Influence of the coating on the Young’s modulus in dependency of the relative coating thickness (**a**) and solid asperity contact pressure versus local fluid film parameter (**b**).

The effects of the coatings on the macro-elastic deformations were taken into account by means of an equivalent Young’s modulus following Liu et al. [88,89]. Thereby, the influence depended on the mechanical properties of the coating and substrate, the coating thickness and the contact conditions. Based upon the mechanical coating properties derived in Part I [73] (see Table 1), the calculated ratios of the Young’s moduli of coated to uncoated UHMWPE, CoCr and Ti64 versus the relative coating thicknesses are displayed in Figure 2a.

Mixed lubrication was taken into account by a statistical approach since a deterministic consideration of surface roughness would require very fine meshing and high computational efforts due to the large contact sizes in soft EHL contacts. Therefore, the Greenwood–Williamson model [90] was utilized and the solid contact pressures *p*_a_ versus the local fluid film parameter:

(12) $$\;\lambda =\frac{h}{\sqrt{R_{q,1}^{2}+R_{q,2}^{2}}}$$

due to the micro-scale deformations of asperities were derived in a preceding simulation in MathWorks MATLAB following [91] and incorporated into the macro-EHL simulation as interpolated functions. The corresponding curves for the pairings studied in this contribution are summarized in Figure 2b.

Finally, the equilibrium of forces between the applied normal load *F* as well as the integral of the total pressure *p*_t_ as sum of hydrodynamic and contact pressure over the contact domain Ω_c_ was ensured by the load balance equation:

(13) $$\;F=\int_{\Omega_{c}}p_{t}\left(x,\;y\right){\;d\Omega }_{c}=\int_{\Omega_{c}}\left[p\left(x,\;y\right){+p}_{a}\left(x,\;y\right)\right]{\;d\Omega }_{c}$$

**Figure 3:**
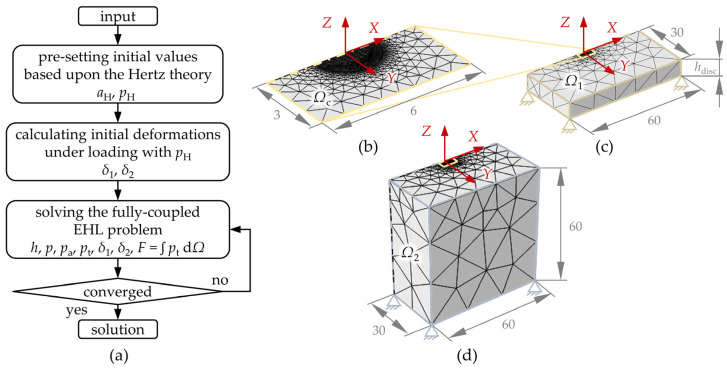
Numerical solution scheme (**a**) and computational domains with meshing and boundary conditions (**b**–**d**).

Numerical modeling was implemented in the software Comsol Multiphysics and the solution scheme is illustrated in Figure 3a. All relevant variables were normalized on Hertzian or initial values:

(14) $$X=\frac{x}{a_{H}},Y=\frac{y}{a_{H}},Z=\frac{z}{a_{H}},P_{t}=\frac{p_{t}}{p_{H}},P=\frac{p}{p_{H}},P_{a}=\frac{p_{a}}{p_{H}},H=\frac{h\cdot R}{a_{H}^{2}},\bar{\rho }=\frac{\rho }{\rho_{0}},\bar{\eta }=\frac{\eta }{\eta_{0}}$$

After initialization with the Hertzian theory and dry conditions, the Reynolds equation was solved in a weak form in the contact domain Ω_c_ (Figure 3b) and strongly coupled with the calculation of the elastic deformations in the domains Ω_1_ (Figure 3c) and Ω_2_ (Figure 3d) using FEM. Thereby, the total contact pressure as normal stress on the top as well as zero displacement were used as boundary conditions. The remaining boundaries featured free boundary conditions with zero normal and tangential stress. The coupling of the solid domains was done by means of linear extrusions. The sizes of the domains were chosen so that the lateral dimensions are large enough for the calculation results to correspond to those of an infinite elastic half-space. For the polymer disk, the influence of the finite height was additionally taken into account. The domains were discretized by tetrahedral meshes with refinements in the contact centers of the upper surfaces and symmetry boundary conditions were applied to reduce the computational effort. More fundamentals about the FEM-based EHL simulation and its implementation can be found elsewhere [83].

## 3. Results

Firstly, the results from the numerical EHL simulation on the initial conditions, and subsequently, the detailed results on the friction and wear behavior from the experimental testing will be shown.

### 3.1. Numerical Results

**Figure 4:**
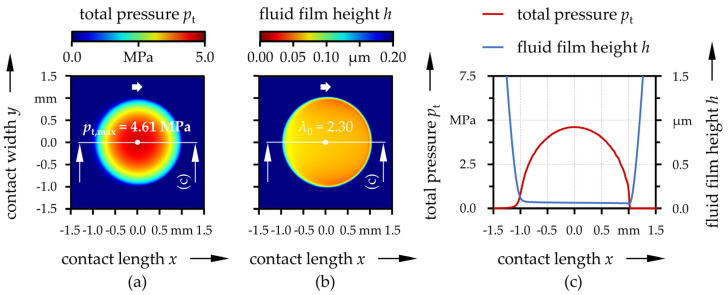
Total pressure (**a**) and lubricant gap (**b**) distribution over the contact area and in the *y* = 0 cross-section (**c**) for the UHMWPE|CoCr-pairing.

The total pressure and lubricant gap distribution over the contact area as well as in the *y* = 0 cross-section are representatively displayed in Figure 4 for the case of the UHMWPE|CoCr-pairing. The other cases were much similar. Basically, the contact displayed typical characteristics for soft EHL contacts [92]. Thereby, the contact center was flattened by elastic deformation with a slight horseshoe-shape and a minimum near the outlet. The pressure followed the Hertzian theory and had a maximum in the contact center. As can be seen in Table 4, *p*_t,max_ was almost the same for all four investigated cases. It falls in the range of 4.59–4.61 MPa independent of the material or coating. The proportions of the load supported by solid asperity contact *F*_a_/*F* as well as values of fluid film parameters in the contact center *λ*_0_ indicated mixed lubrication conditions for the uncoated cases (Table 4). With the coatings, the solid asperity load carrying ratios were significantly increased. However, as the coating thicknesses are very low compared to the elastically deformed contact area, this was less due to the mechanical coating properties but rather due to the higher roughness, especially of UHMWPE:H.

**Figure 5:**
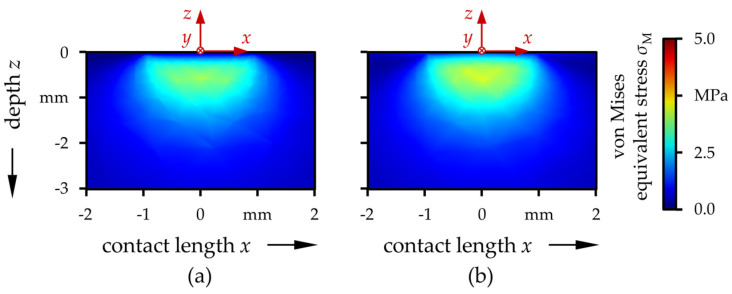
Calculated von Mises equivalent stress distribution in the *y* = 0 cross-section plane of the UHMWPE disk (**a**) and the CoCr pin (**b**).

Furthermore, the distribution of the von Mises equivalent stresses in the component depth direction of the *y* = 0 plane are exemplarily shown for UHMWPE and CoCr in Figure 5. The other three cases again showed a strong similarity. The stress field generally featured typical characteristics for Hertzian or EHL contacts [93,94]. For the compared load case, geometries and material properties, with the resulting large contact dimensions, the maximum values were considerably beneath the surface and also for the coated specimens well away from the coating/substrate interface.

### 3.2. Experimental Results

**Figure 6:**
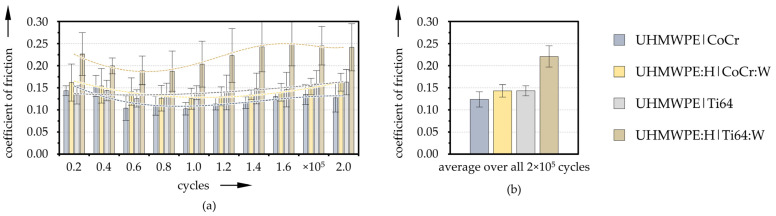
Evolution of the mean friction coefficients for each testing interval and trend lines (**a**) as well as averaged values over the entire test duration (**b**) for the reference and coated pairings (*n* = 3).

The coefficient of friction was measured in-situ during the tests. The mean values and the standard deviations for each of the testing intervals as well as the average over the entire tests for the reference and coated pairings are depicted in Figure 6. After initially slightly elevated values, the friction coefficients for all pairings dropped slightly with running-in and rose again with increasing wear and test duration (Figure 6a). From each interval, and also from the total average (Figure 6b), it can be seen that the COF of the pairings with CoCr exhibited slightly lower values than with Ti64. In addition, the coated specimens showed higher friction than the uncoated references.

**Figure 7:**
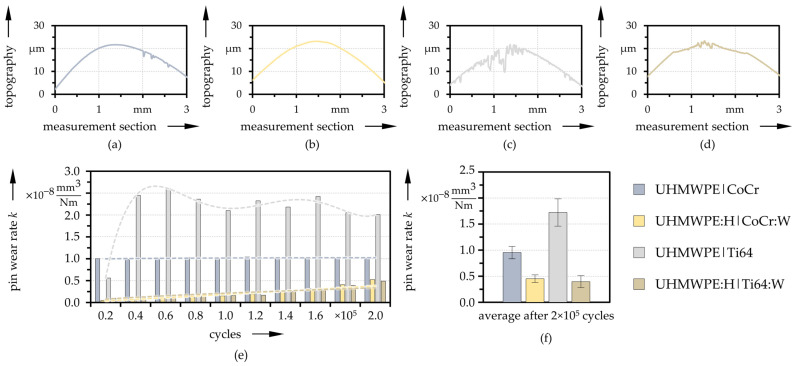
Exemplary cross sections of the pins (**a**–**d**) as well as evolution of the pin wear coefficients with trend-lines (*n* = 1) after each testing interval (**e**) and averaged values (*n* = 3) after the entire test duration (**f**) for the reference and coated pairings.

Representative topographies of the pin surface after 2 × 10^5^ cycles, captured by LSM, are exemplarily plotted in Figure 7. The reference pins showed no pronounced wear calotte but pronounced scratches and grooves over the entire contact surface, which was more distinct for Ti64 (Figure 7c) than for CoCr (Figure 7a). The extent of the wear marks, however, can only be recognized to a limited extent from the cross-sections. On the contrary, the coated pins (Figure 7b,d) did not display as clear scores, but rather a uniform wear of the coating. The area affected by wear was also considerably smaller. Accordingly, the calculated wear rates differed from those of the references as can be seen in Figure 7e,f), where the wear rate evolution for one specimen per pairing evaluated after each interval, as well as the values averaged over all specimens after the full testing time are compared. While the uncoated references exhibited rather constant and higher values over the test intervals without a clear trend, the coated pins featured an upward trend (Figure 7e). Both CoCr:W and Ti64:W showed very low wear rates at the beginning, which progressively increased during the test as the functional coating began to degrade in the highest stressed pin center. Still, the wear rate remained beneath that of the references. In average, Ti64 shows the highest wear, followed by the CoCr reference (Figure 7f). The coatings led to a wear reduction of roughly 49% (CoCr:W) and 77% (Ti64:W) on average. Here, the coatings on both substrates were fairly similar.

**Figure 8:**
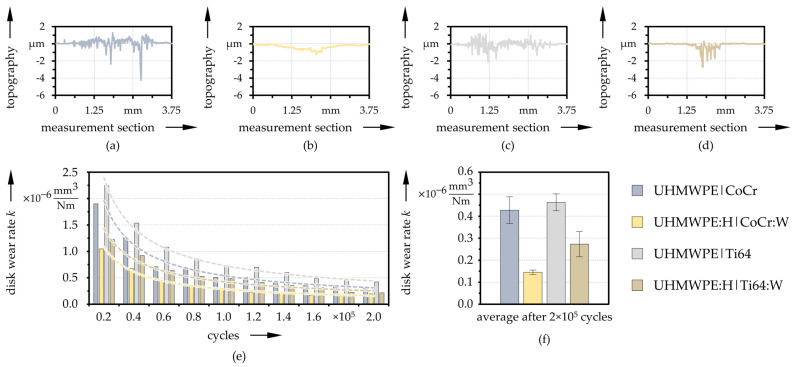
Exemplary cross sections of the wear track on the disks (**a**–**d**) as well as evolution of the disk wear coefficients with trend-lines (*n* = 1) after each testing interval (**e**) and averaged values (*n* = 3) after the entire test duration (**f**) for the reference and coated pairings.

While the uncoated disks showed adhesive and abrasive wear in particular, the coated disks showed more plastic deformation at the edges of micro-ploughed ridges. Some representative surface topographies across the wear track of the four variants after 2 × 10^5^ cycles are exemplarily shown in Figure 8a–d. The evolution of the wear rate during each interval, as well as after the full test are compared in Figure 8e,f, respectively for one representative specimen per pairing and averaged over all specimens. The wear rate was observed to decrease throughout a single test. Compared to the uncoated reference specimens, the coatings resulted in a significant reduction of the wear rate; in the case of the CoCr:W counter-body by 66% and for the pairing with Ti64:W by 41%. In addition, the pairings with Ti64 or Ti64:W tended to exhibit slightly higher wear of the disks than with CoCr or CoCr:W, respectively. Besides the material, the differences in wear rates between the pin and the disk can be attributed to the contact arrangement. While the top of the pin was always in contact, a volume element of the disk was only slid over once per revolution.

**Figure 9:**
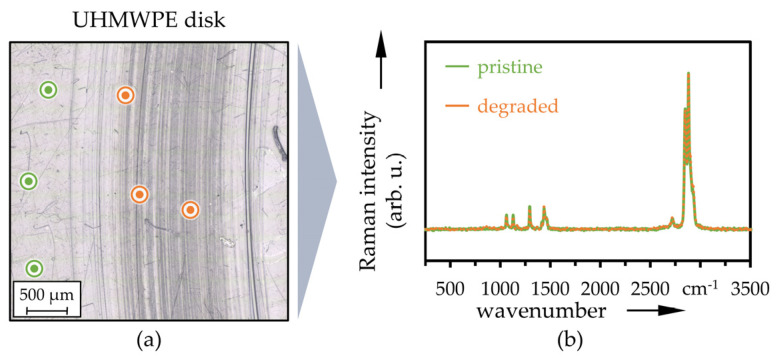
LM image with Raman measurement positions (**a**) and averaged Raman spectra (**b**) at pristine and degraded positions of the UHMWPE disk after sliding against the CoCr pin (*n* = 3).

**Figure 10:**
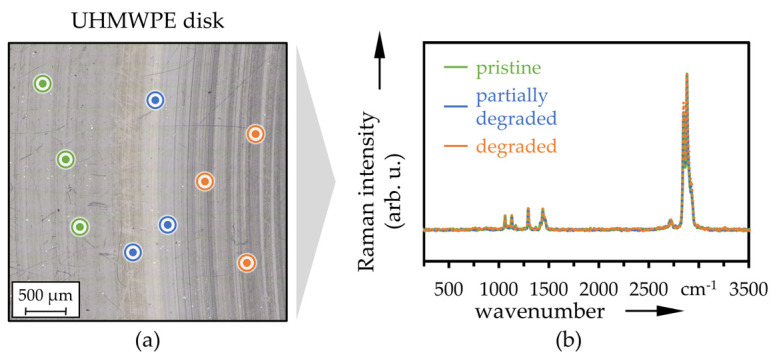
LM image with Raman measurement positions (**a**) and averaged Raman spectra (**b**) at pristine and degraded positions of the UHMWPE disk after sliding against the Ti64 pin (*n* = 3).

Corresponding LM images of the wear track on UHMWPE reference disks after 2 × 10^5^ cycles with measurement positions of Raman spectroscopy (a) as well as averaged spectra (b) at locations outside (pristine, green color) and inside the wear track (degraded, orange) as well as in between (partially degraded, blue) are displayed in Figure 9 (versus CoCr) and Figure 10 (versus Ti64), respectively. With small peaks at 1065, 1293, 1305, 1435 and 2724 cm^−1^ as well as pronounced peaks at 2848 and 2880 cm^−1^, this showed typical characteristics of UHMWPE [73,95,96] and no changes due to tribological testing and wear. The CoCr and Ti64 counter-bodies are not Raman active [73], and therefore, no results could be shown here.

**Figure 11:**
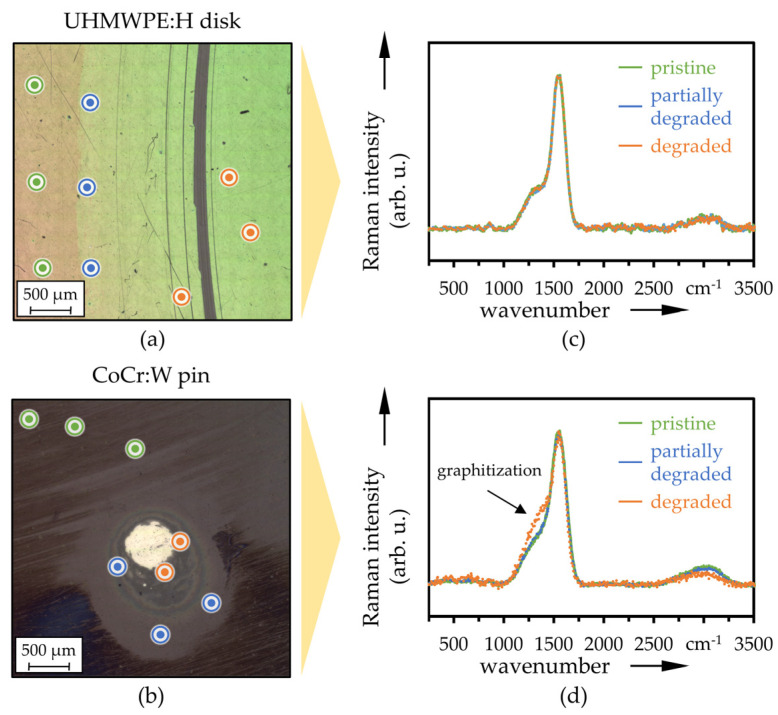
LM images with Raman measurement positions on the disk (**a**) and pin (**b**) as well as averaged Raman spectra (*n* = 3) at pristine, partially degraded and degraded positions of UHMWPE:H (**c**) and CoCr:W (**d**).

**Figure 12:**
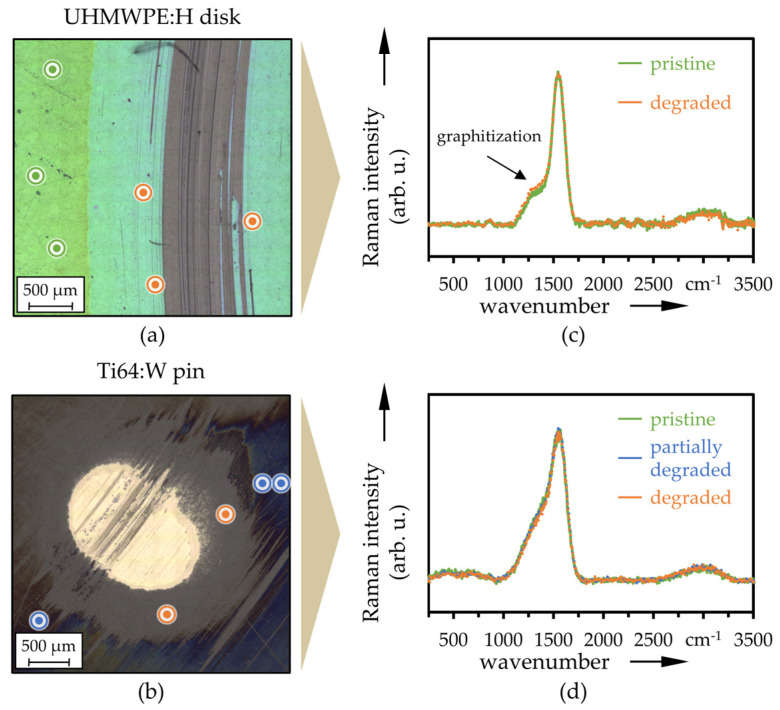
LM images with Raman measurement positions on the disk (**a**) and pin (**b**) as well as averaged Raman spectra (*n* = 3) at pristine, partially degraded and degraded positions of UHMWPE:H (**c**) and Ti64:W (**d**).

LM images of the wear marks on the coated disks (a) and the wear calotte on the pins (b) after 2 × 10^5^ cycles are depicted in Figure 11 (UHMWPE:H|CoCr:W) Figure 12 (UHMWPE:H|Ti64:W), respectively. Therein, the positions of the averaged Raman signals plotted in Figure 11c,d and Figure 12c,d are also mapped. The pristine Raman spectra on the disks and pins featured pronounced peaks around 1350 cm^−1^ and 1560 cm^−1^, representing typical centers for the D- and G-bands of DLC coatings. In addition, a slightly noticeable peak was present around 850 cm^−1^, which was due to trans-polyethyne [97,98,99]. The broad and slightly distinct peak in the region of high wavenumbers between 2700 cm^−1^ and 3200 cm^−1^ could be attributed to 2D and 2G bands, overlapped by symmetric and anti-symmetric stretching modes due to the hydrogen incorporation [100]. The ratios of the intensities of the D- and G-band (*I*_D_/*I*_G_) were calculated to be in the range of 0.2 and 0.3, which are also characteristic values for hydrogenated amorphous carbon coatings [99,101].

The centers of the wear calotte on the CoCr:W and Ti64:W pins were not Raman active (Figure 11b,d and Figure 12b,d), indicating metallic adhesion or support layer as well as a fully worn DLC layer. This is also due to the fact that they are in contact and stressed during the entire test period. Conversely, with the exception of some scratches, the UHMWPE:H disks exhibited a clear DLC Raman signal also in large parts on the wear track (Figure 11a,c and Figure 12a,c). The surface in the wear track of the disks and also at the edge of the wear calottes of the pins was affected by the tribological stress and experienced certain graphitization, which is reflected in the slightly enhanced D-peak (higher *I*_D_/*I*_G_ ratio) and the lightly lowered 2D- and 2G-peak, (see for example Figure 11d and Figure 12a).

**Figure 13:**
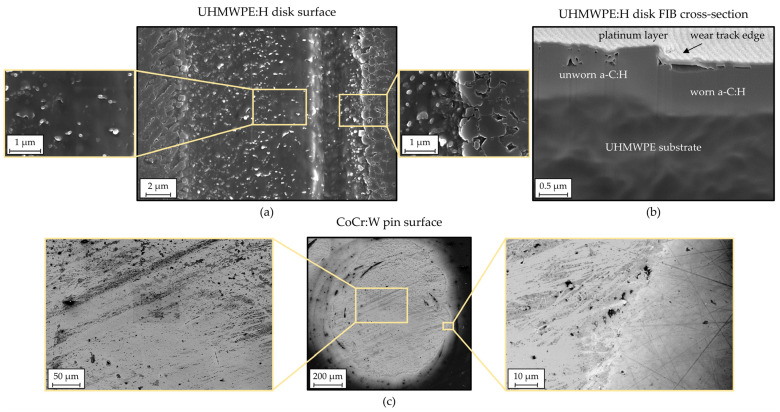
SEM micrographs of the wear track (**a**) and a FIB cross-section at the wear track edge (**b**) of the UHMWPE:H disk and SEM micrographs of the worn CoCr:W pin surface (**c**).

The wear on the disk and pin also becomes evident in the SEM images, as exemplified for a UHMWPE:H|CoCr:W pairing after 2 × 10^5^ cycles in Figure 13. While the pin showed apparent coating deterioration and the substrate could be recognized (Figure 13c), the disk displayed a wear track, but was still covered by a homogeneous coating and only featured slight fractures in the edge area. From the FIB cross-section of the transition between the wear track and the pristine surface depicted in Figure 13c, it can be seen that the coating was still intact and experienced continuous wear without signs of spalling or crack initiation. In addition, some permanent compaction of the UHMWPE substrate can be observed, which also caused the coating to sag. However, the latter was able to withstand this strain. The actual reduction of the coating thickness could be estimated from the FIB cross-section image to be merely around 200 nm.

**Figure 14:**
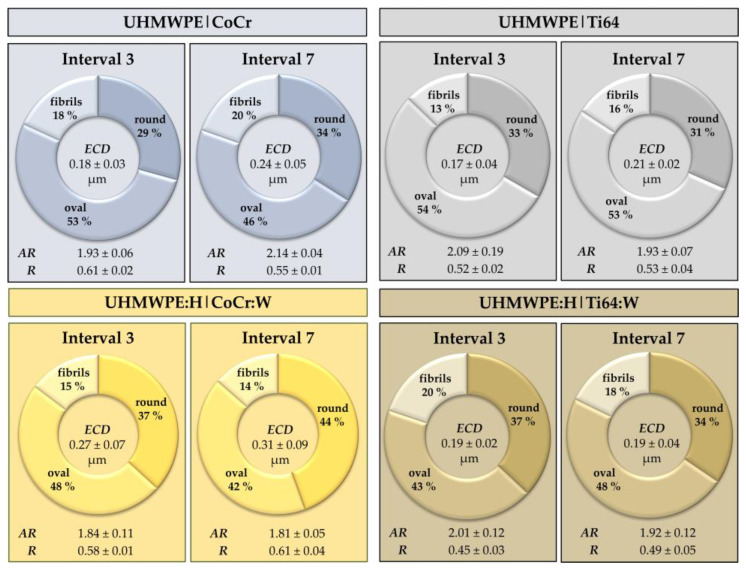
Mean values and standard deviations of *ECD*, *AR* and *R* as well as the portions of round, oval and fibril-like particles for the four pairings at two analyzed intervals (3 and 7) of the tribometer tests.

The results of the particle analyses including *ECD*, *AR* and *R* are summarized in Figure 14, whereby the respective portions of round, oval and fibril-like particles according to Catelas et al. [102] are also given. A *t*-test for dependent groups revealed a significant difference in particle size (*ECD*) from Interval 3 to Interval 7 for the UHMWPE|CoCr (*p* = 0.006) and the UHMWPE|Ti64 pairing (*p* = 0.026). No significant differences in the particle sizes were found for the UHMWPE:H|CoCr:W (*p* = 0.535) and the UHMWPE:H|Ti64:W pairing (*p* = 0.819). Regarding the *AR*, no substantial differences were found from Interval 3 to Interval 7 for all groups (*p* > 0.283). However, regarding the *R*, a significant difference was found for the UHMWPE:H|Ti64:W pairing from Interval 3 to Interval 7 (*p* = 0.035) while no considerable differences were found for the other three groups (*p* > 0.237). For the group comparison of the Interval 7 using ANOVA for independent testing and Bonferroni post-hoc analysis, a significant difference was found for the *ECD* (*p* = 0.007) and for the *R* (*p* = 0.038) between the two coated groups, but no significant difference was observed for the *AR* (*p* = 1.000). Between the two non-coated reference groups, no significant difference was noticeable (*p* = 1.000). Furthermore, no major difference was found for any other group comparison of the three parameters (*p* > 0.258), except for the comparison between the UHMWPE:H|CoCr:W pairing and the UHMWPE|Ti64 pairing (*p* = 0.026) regarding the *ECD*.

## 4. Discussion

### 4.1. Relevance of the Experimental Test Conditions

The contact and lubrication conditions in the studied pin-on-disk model tests generally featured similarities to those observed in some steps of a transient load collective from the ISO 14243 [47,49] gait cycle, see Marian et al. [67,68]. In particular, the contact dimensions and deformations of the rubbing surfaces observed here matched well instants with low fluid film heights near the reversal points of motion/velocities. Yet, the pressures that occurred in the setup of the present investigations were in the lower range of typical stress collectives for TKAs. For UHMWPE, however, these moderate values were reported to be more wear-critical than significantly higher or lower pressures [72,103]. It was also predicted by the simulation and confirmed by the experimentally determined *COF* values that pronounced mixed lubrication conditions were present. This was even intensified due to the slightly higher roughness of the coated surfaces compared to the references and typical roughness values of knee replacements before implantation [104,105]. Therefore, it can be concluded that the experimental test conditions were suitable for analyzing the wear behavior for the investigated reference and coated pairings under harsh conditions and to ensure a certain transferability to a subsequent application on real TKA components.

### 4.2. Friction and Wear Behavior

Compared to the references, coating resulted in higher friction. This has also been reported for DLC coatings in other publications, for example [35,58]. Since the nominal contact area did not experience relevant differences, this can be partly attributed to the stronger adhesion tendency of chemically similar surfaces, and in particular, to the higher roughness and thus, the deteriorated lubrication conditions of the DLC-coated pairings, as verified by numerical modeling. In principle, this alone does not necessarily represent a significant disadvantage. However, the influence of friction-induced heating in the contact area (flash temperatures) and limited heat dissipation [106] on the degeneration of UHMWPE or the denaturation of the SF due to exceeding the coagulation temperatures of proteins [107] should be considered carefully. These effects could also be limited to some extent by a further polishing step and thus a reduction in the roughness of the coated surfaces.

Despite the increase in friction, DLC-coating the contacting partners led to a considerable reduction of the wear volume of the UHMWPE substrate. No delamination or spalling of larger wear particles occurred, due to the good compliance with macro-elastic deformation of the substrates and at the same time very good adhesion (see Part I [73]) of the thin coatings in combination with the highest stresses clearly below the layer/substrate-transition. This may be attributed to the comparatively soft coating, which is capable of withstanding the tribological stresses by continuous, yet slow wear. Thus, the coating provided suitable protection to the metallic and UHMWPE substrates, where wear has been significantly reduced. Due to the UHMWPE compression and sagged coating, the determined wear rates were rather conservative and overestimated the actual coating wear. Unlike other examples reported in literature, e.g., [43,44], the wear was reduced even though the contact was lubricated by artificial SF (BS) and boundary absorption layer of proteins could potentially form. The analyzed particles of the four groups revealed that the UHMWPE:H|CoCr:W pairing released the biggest sized particles. However, the particles were still in the nanometer range and stayed similar from Interval 3 to Interval 7. The particle size of the UHMWPE:H|Ti64:W pairing was similar to that of the reference groups and did not change from Interval 3 to Interval 7 too. These results indicated that the coated groups did not suffer from any delamination, which would result in an increased wear from Interval 3 to Interval 7 with particles of several micrometers in size. In contrast, the particle sizes of the two reference pairings increased from Interval 3 to Interval 7. As the released particles for the coated pairings were clearly distinguishable from UHMWPE particles, it could be estimated that primarily amorphous carbon particles of the different coatings and possibly metallic or metallic-carbide particles of CoCr:W and Ti64:W were released. Due to the promising biocompatibility of the amorphous carbon itself [73] and the small particle sizes, this can be considered less critical than delamination or flaking of larger coating segments or pure UHMWPE wear. This distinguishes the DLC coatings studied within this contribution from coatings based upon tetrahedral amorphous carbon coating studied in literature, where, despite good wear resistance, ablation of larger wear particles is more likely to occur due to the high hardness, induced stresses and different deformability between substrate and coating.

### 4.3. Applicability and Limitations

This two-part study constituted a first step towards the deposition of amorphous carbon coatings on implant materials with excellent mechanical properties and advantageous tribological behavior. Limitations naturally arise from the pin-on-disk model setup with simplified geometries and pure sliding conditions. With beneficial coating architectures being identified, the next step is to further investigate them in tests with more complex dynamics, such as rolling-sliding test-rigs, as well as real components on knee simulators. In addition, the tribological behavior was solely investigated for a standardized artificial SF based upon diluted BS. The possible influence of various constituents of the SF is to be investigated, especially for hyaluronic acid (HA). The latter can play a crucial role for synovial joint tribology [68,71] and rheology [108] but was no constituent of the lubricant used in this study. However, the poor fluid film formation with predominantly mixed and boundary lubrication was a result of the low viscosity, which was in the range of human joints affected by inflammatory disease [77], and can be interpreted as a kind of worst-case scenario that the coatings had to withstand. Ultimately, it should be noted that UHMWPE is increasingly being replaced by HXLPE [9]. It is expected by the authors, though, that this will have no major impact on the mechanical and adhesion properties as well as the tribological behavior of the amorphous carbon coatings under investigation. Furthermore, the particle sizes and morphologies of the studied pairings might demand for further investigations, for instance in a multidirectional test-setup, to determine whether cross shearing would lead to delamination and/or the release of bigger particles.

## 5. Conclusions

This contribution evaluated the potential of amorphous carbon coatings for improving the tribological behavior of TKAs. While Part I [73] addressed the deposition of a-C:H:W and a-C:H coatings by PVD as well as the biochemical and mechanical properties, this study focused on elaborating the tribological behavior using model tests with CoCr and Ti64 pins on UHMWPE disks and diluted BS lubrication. Numerical EHL contact simulation was used to assess the representativity for TKAs and the influence of coatings on contact and lubrication conditions. The wear behavior was analyzed by means of LM, LSM, SEM and Raman spectroscopy. For the studied coatings, materials and contact conditions, the following conclusions could be drawn:

- Representative conditions for the application could be established in model tests. This allowed, in particular, an initial screening for surface modifications as well as the isolated observation of wear phenomena. Nevertheless, the subsequent transfer to component tests remains essential.
- The considered thin coatings did not lead to any significant changes in the macro-geometrical elastic deformation behavior and contact area of TKAs.
- Higher surface roughness of the coated specimens led to an increase in friction compared to uncoated references. Further process steps to smoothen the coated surface, either on the process side or through a subsequent polishing step, could be desirable.
- Despite the higher friction, amorphous carbon coatings contributed to significant wear reduction on the CoCr and Ti64 pins as well as the UHMWPE disks in particular. Thereby, the DLC coatings exhibited no delamination or spalling of larger wear particles but rather continuous and slow wear rates. Thus, the UHMWPE disk was still protected by an intact coating without signs of cracks and fatigue even after the complete testing period. It can be hypothesized that the service life of the tibial inlay is extended by the durability of the coating.
- The wear of the DLC-coated Ti64 pairing was below the CoCr reference. Therefore, Ti64 with an amorphous carbon coating bears the potential to supplant CoCr as implant material.
- It can be assumed that—if the adhesion and wear resistance are sufficiently high—amorphous carbon coatings as studied within this contribution can lead to a reduction of UHMWPE wear particle-induced aseptic loosening of TKAs.

## Figures and Tables

**Table 1:** Averaged key properties of the substrates and amorphous carbon coatings as determined in [73].

	UHMWPE	UHMWPE:H	CoCr	CoCr:W	Ti64	Ti64:W
coating thickness *t*_c_	-	1.4 μm	-	1.0 μm	-	1.2 μm
arithmetical mean roughness *R*_a_	0.02 μm	0.05 μm	0.02 μm	0.02 μm	0.03 μm	0.03 μm
root-mean-squared roughness *R*_q_	0.03 μm	0.06 μm	0.02 μm	0.03 μm	0.04 μm	0.03 μm
indentation hardness *H*_IT_	0.05 GPa	1.3 GPa	11.6 GPa	16.1 GPa	5.3 GPa	14.4 GPa
indentation modulus *E*_IT_	0.56 GPa	4.25 GPa	252 GPa	148 GPa	132 GPa	153 GPa
hardness/modulus *H*_IT_/*E*_IT_	0.085	0.309	0.046	0.097	0.040	0.106
critical normal load *L*_c3_	-	77 N ^1^	-	22 N ^2^	-	21 N ^2^

^1^ Performed with 100Cr6 ball indenter (*r* ≈ 2 mm); ^2^ performed with Rockwell diamond indenter.

**Table 2:** BCS composition as specified by the supplier [77].

Parameter/Biochemical Assay		Electrolytes	
alk. phosphatase	151 U/L	sodium	147 mmol/L
GOT (AST)	79 U/L	potassium	6.7 mmol/L
GPT (ALT)	37 U/L	calcium	1.95 mmol/L
γ-GT	41 U/L	magnesium	0.91 mmol/L
bilirubin total	3.42 µmol/L	phosphate	2.29 mmol/L
LDH	1345 U/L	iron	15.2 µmol/L
CK total	359 U/L	**Capillary Electrophoresis**	
cholesterol	3.57 mmol/L	sodium	147 mmol/L
triglycerides	0.18 mmol/L	potassium	6.7 mmol/L
creatinine	106.1 µmol/L	calcium	1.95 mmol/L
urea	5.83 mmol/L	magnesium	0.91 mmol/L
glucose	6.11 mmol/L	phosphate	2.29 mmol/L
protein	72 g/L	iron	15.2 µmol/L

**Table 3:** Rheological properties of the substitute SF in accordance to [77].

Zero shear viscosity *η*_0_	30 mPa∙s
Infinite shear viscosity *η*_∞_	0.8 mPa∙s
Cross time constant *α*	11 s
Cross rate constant *m*	0.73
Base density *ρ*_0_	1000 kg/m³

**Table 4:** Computed maximum total contact pressure, solid asperity load share and central fluid film parameter for the reference as well as the coated pairings.

	UHMWPE|CoCr	UHMWPE|Ti64	UHMWPE:H|CoCr:W	UHMWPE:H|Ti64:W
max. total pressure *p*_t,max_	4.61 MPa	4.60 MPa	4.60 MPa	4.59 MPa
solid asperity load share *F*_a_/*F*	7.5%	19.4%	81.3%	81.6%
central fluid film parameter *λ*_0_	2.30	1.87	1.93	1.92

## Data Availability

The data presented in this study are available on request from the corresponding authors.

## Nomenclature

*a*_H_	Hertzian contact width
*A*_c,1_	cross-sectional area
*AR*	particle aspect ratio
*c*	circumference of the wear track
*C*	compliance matrix
*COF*	coefficient of friction
*ECD*	equivalent particle circle diameter
*E*_i_	Young’s modulus
*E*_IT_	indentation modulus
*F*	normal load
*h*	lubricant gap
*h*_0_	rigid body distance
*h*_disk_	height of the UHMWPE disk
*h*_s,i_	score height
*H*	normalized lubricant gap
*H*_IT_	indentation hardness
*I*_D_	D-band Raman intensity
*I*_G_	G-band Raman intensity
*k*	wear rate
*l*_s,i_	score length
*m*	cross rate constant
*p*	hydrodynamic pressure
*p*_a_	solid asperity pressure
*p*_H_	maximum Hertzian contact pressure
*p*_t_	total contact pressure
*P*	normalized hydrodynamic pressure
*P*_a_	normalized solid asperity pressure
*P*_t_	normalized total contact pressure
*r*_c_	curved wear calotte radius
*R*	pin radius
*R*	particle roundness
*R*_i_	worn pin radii
*R*_q,i_	root-mean-squared roughness
s	sliding distance
*t*_c_	coating thickness
*t*_c,i_	worn off coating thickness
*u*	sliding velocity
*U*	displacement vector
*U_z_*	displacement in z-direction
*W*_acc_	accumulated wear volume
*W*_p_	micro-ploughed wear volume
*W*_v_	volumetric wear
*x, y, z*	cartesian space coordinates
α	cross time constant
$\dot{\gamma }$	shear rate
*δ_i_*	elastic deformation
*ε*	strain tensor
*η*	viscosity
*η_0_*	zero shear viscosity
*η_∞_*	infinite shear viscosity
$\bar{\eta }$	normalized viscosity
*θ*	fractional film content
*λ*	fluid film parameter
*ν*	Poisson’s ratio
*ρ*	density
*ρ_0_*	base density
$\bar{\rho }$	normalized density
*σ*	stress tensor
Ω_1_	computation domain disk
Ω_2_	computation domain pin
Ω_c_	computation domain contact area
